# Managing hydrocephalus in patients with leptomeningeal disease: A multicenter retrospective analysis

**DOI:** 10.1002/ijc.35505

**Published:** 2025-06-04

**Authors:** Obada T. Alhalabi, Lukas Klein, David Wasilewski, Amine Mellal, Carmen Büsken, Clara Buszello, Giulia Cossu, Ilker Y. Eyüpoglu, Andreas W. Unterberg, Peter Vajkoczy, Gabriele Schackert, Mahmoud Messerer, Martin Misch, Tobias Kessler, Wolfgang Wick, Christine Jungk, Ahmed El Damaty, Sandro M. Krieg, Tareq A. Juratli, Alexander Younsi

**Affiliations:** ^1^ Department of Neurosurgery Heidelberg University Hospital Heidelberg Germany; ^2^ Medical Faculty, Heidelberg University Heidelberg Germany; ^3^ Department of Neurosurgery Charité—Universitätsmedizin Berlin, corporate member of Freie Universität Berlin, Humboldt‐Universität zu Berlin, Berlin Institute of Health Berlin Germany; ^4^ Department of Neurosurgery University Hospital of Lausanne and University of Lausanne Lausanne Switzerland; ^5^ Department of Neuroscience University Hospital of Lausanne and University of Lausanne Lausanne Switzerland; ^6^ Department of Neurosurgery University Hospital Carl Gustav Carus, Technische Universität Dresden Dresden Germany; ^7^ Clinical Cooperation Unit Neurooncology, German Cancer Research Center (DKFZ) Heidelberg Germany; ^8^ Department of Neurology and Neurooncology Program National Center for Tumor Diseases, Heidelberg University Hospital Heidelberg Germany

**Keywords:** CSF diversion, intrathecal therapy, leptomeningeal disease, Rickham reservoir, ventriculoperitoneal shunt

## Abstract

Leptomeningeal disease (LMD) represents a terminal condition of tumor cell seeding that can cause symptomatic hydrocephalus. With improved survival rates under systemic therapy, the role of cerebrospinal fluid (CSF) drainage through ventriculo‐peritoneal shunt (VPS) or Rickham reservoir (RR) placement in LMD patients is gaining more relevance. This study aimed to compare outcomes of both modalities in a multicentric contemporary cohort. A retrospective analysis of medical charts in patients receiving VPS for LMD and malresorptive hydrocephalus in two neurosurgical centers between 2006 and 2021 yielded 64 patients. The most common underlying oncological conditions were breast (*n* = 32, 49%) and non‐small cell lung cancer (NSCLC, *n* = 16, 25%). The median time between primary and LMD diagnosis was 23.3 months (11.2 to 43.4 months). Symptoms of intracranial hypertension were relieved in 79% of cases (*n* = 50) after shunting, with 42 (66%) and 32 patients (50%) receiving systemic and intrathecal therapy, respectively. A further multicenter analysis comparing patients receiving VPS with patients receiving RR (with regular tapping) included 155 patients (VPS: *n* = 80, 52%; RR: *n* = 75, 48%). Compared to VPS, RRs were associated with a lower surgical revision rate (8% vs. 24%, *p* = 0.009). There was no difference in median overall survival in VPS patients (118 days) compared to RR patients (80 days, *p* = 0.180). Given this data showing a short and comparable survival of patients under both modalities with a lower RR complication rate, a rationale for an initial Rickham implantation in LMD patients with hydrocephalus, with later VPS conversion for long‐term surviving patients, could be contemplated.

## What's New?

Leptomeningeal disease is a highly palliative condition of tumor cell seeding that can cause symptomatic hydrocephalus. This multi‐center retrospective study of patients with leptomeningeal disease‐associated hydrocephalus compared the placement of ventriculoperitoneal shunts and Rickham reservoirs for cerebrospinal fluid drainage. No significant difference was found in median overall survival between the two groups, with a significantly lower complication rate for Rickham reservoirs. As intrathecal therapy use and patient survival increase, this study aids in assessing the risks and benefits of both modalities, providing a rationale for initially implanting Rickham reservoirs and offering ventriculoperitoneal shunts conversion as a later option.

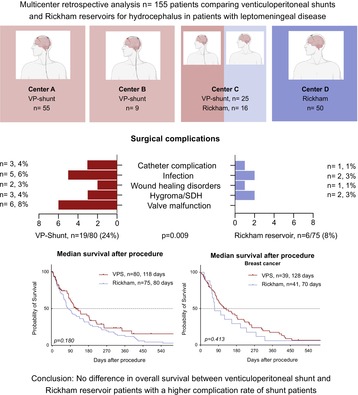

**Abbreviations**
ALK: Anaplastic Lymphoma Kinase
BM: Brain Metastasis
CHUV: Centre Hospitalier Universitaire Vaudois
CNS: Central Nervous System
CSF: Cerebrospinal Fluid
CUP: Cancer of Unknown Primary
ECOG: Eastern Cooperative Oncology Group
EGFR: Epidermal Growth Factor Receptor
ER: Estrogen receptor
HER2: Human Epidermal Growth Factor Receptor 2
IQR: Interquartile Range
KPS: Karnofsky Performance Scale
LMD: Leptomeningeal Disease
LP: Lumbar Puncture
MRI: Magnetic Resonance Imaging
NSCLC: Non‐Small Cell Lung Cancer
OS: Overall Survival
PR: Progesterone receptor
RR: Rickham Reservoir
TNM: Tumor, Node, Metastasis (staging system)
VPS: Ventriculoperitoneal Shunt

## 1 INTRODUCTION

Leptomeningeal disease (LMD) describes the seeding of tumor cells within the leptomeninges and the subarachnoid space, including CSF (cerebrospinal fluid) spaces. It affects 3% to 5% of patients with solid tumors and up to 20% in autopsy studies and involves mainly breast, non‐small cell lung cancer (NSCLC) and melanoma patients.^1^, ^2^ After LMD diagnosis, the overall survival rate ranges between 3 and 4 months and is influenced by the status of primary disease, number of brain metastases, intrathecal therapy, and the general patient condition, represented by the Karnofsky Performance Scale (KPS) or Eastern Cooperative Oncology Group (ECOG)‐status.^3^, ^4^, ^5^, ^6^ The current possibilities of LMD treatment are heterogeneous and include (targeted) systemic therapies, intrathecal chemotherapy (with the intraventricular route via ventricular access devices preferred to the lumbar) for non‐adherent disease^7^, ^8^ experimental intrathecal antibody treatments,^9^ and irradiation.^2^, ^10^, ^11^ Considering the fact that the outcome of patients with brain metastasis (BM) is limited by systemic disease,^11^, ^12^, ^13^ LMD therapy aims at delaying neurological deterioration and prolonging survival with an acceptable quality of life for affected patients.^14^

Symptomatic malresorptive hydrocephalus and subsequent intracranial hypertension are a result of LMD and could cause a drastic deterioration of the neurological status of affected patients, warranting prompt treatment.^15^, ^16^ Previous studies demonstrated the benefits of CSF diversion via a ventriculoperitoneal shunt (VPS) in terms of symptom relief, hospital discharge, and the possibility of intrathecal therapy, which in turn has been shown to improve survival and delay neurological deterioration.^17^, ^18^, ^19^, ^20^, ^21^ The spectrum of the usually frequent postoperative complications of VPS comprises surgical site infection, subdural hygroma, and hematoma, valve dysfunction, and abdominal catheter complications.^22^ Outcomes after shunting are reported to be better in groups of patients with controlled disease, good general condition, and brain oligo‐metastases.^23^, ^24^

Given the prolonged survival in patients with (especially human epidermal growth factor receptor 2‐postive, HER2+) breast cancer and NSCLC with epidermal growth factor receptor (EGFR) mutations and anaplastic large‐cell lymphoma kinase (ALK) rearrangements over the past years due to improvements in targeted therapies, the treatment of hydrocephalus associated with LMD through CSF drainage via VPS has experienced an increasing relevance.^25^, ^26^, ^27^, ^28^ Owing to a potentially higher complication rate and the comparatively short survival of VPS patients with LMD, the idea of Rickham reservoir (RR) implantation for simultaneous intrathecal therapy and CSF drainage is entertained by many centers, which could pose an alternative strategy.^29^, ^30^

We hence explored the outcomes of patients receiving VPS implantation for symptoms of malresorptive hydrocephalus in a bicentric analysis. By pooling data from four centers, we then contextualized this analysis to compare the surgical and survival outcomes of patients with LMD receiving VPS and patients receiving RR only.

## 2 METHODS

### 2.1 Study design and data collection: Bicentric cohort

We performed a bicentric, retrospective analysis of all consecutive patients undergoing ventriculoperitoneal shunt (VPS) implantation for LMD of extracranial solid primary disease between 2006 and 2021 in Center A (Department of Neurosurgery at the University Hospital Heidelberg) and between 2010 and 2023 in Center B (Department of Neurosurgery of the Centre Hospitalier Universitaire Vaudois [CHUV], Lausanne, Switzerland). Both centers pursue a similar treatment strategy for patients with hydrocephalus due to LMD using VPS. Patients with occlusive hydrocephalus and/or LMD from primary brain tumors, lymphoma, or leukemia were excluded. Demographic, clinical, radiological, and histopathological data of the primary tumor were extracted from medical records including age at diagnosis, patients' history, and clinical presentation including the presence of pre‐operative hydrocephalus, primary cancer, and number/localization of brain metastases. Furthermore, surgical data such as implanted VPS valves, peri‐surgical complications (VPS obstruction or failure, VPS infection, VPS over‐drainage, peritoneal carcinomatosis, generalized infection, hemorrhage, wound healing disorder) and surgical revisions were assessed. Postoperative neurological outcomes, CSF findings after lumbar puncture (opening pressure, protein, lactate, glucose, cell number and cell morphology as well as histopathological diagnosis), adjuvant treatment, hospital discharge, and patient survival were also analyzed. The pre‐ and postoperative neurological symptoms and the general condition of patients (using the Karnofsky Performance Score (KPS) as a surrogate) were analyzed.

### 2.2 Multicenter analysis

Data from two additional centers was pooled with the bicentric analysis data: Center C (Department of Neurosurgery, Charité, Berlin, Germany), and Center D (Department of Neurosurgery, University Hospital Carl Gustav Carus, Technische Universität Dresden, Dresden, Germany). In general, centers usually applied either procedure based on their treatment philosophy for LMD patients with hydrocephalus: Centers A and B implant VPS, Center C implants RR and VPS, while Center D almost exclusively implants RR with regular drainage punctures for LMD as an institutional treatment strategy, even in patients with hydrocephalus. VPS patients were then compared to RR patients in terms of age, primary disease, surgical procedure complications, and survival rates.

### 2.3 Statistical analysis

Patient characteristics were analyzed using descriptive statistics. Continuous variables are reported as medians and interquartile ranges (IQR) if not stated otherwise. Ordinal and nominal variables are presented as numbers and frequencies. All statistical comparisons were performed using PRISM (Version 10) and *p* < 0.05 was regarded as significant. Nominal variables between groups were compared using Chi‐Square or Fisher's exact tests (depending on group size). The Mann–Whitney test was used for non‐parametric continuous data whereas parametric tests on normally distributed data were carried out using the double‐tailed Student's *t*‐test (in non‐paired samples). Data distribution was assessed using the Kolmogorov–Smirnov test. The Log‐rank (Mantel‐Cox) test was used for survival analyses.

## 3 RESULTS

### 3.1 Clinical characteristics of patients with LMD receiving VPS

**FIGURE 1:**
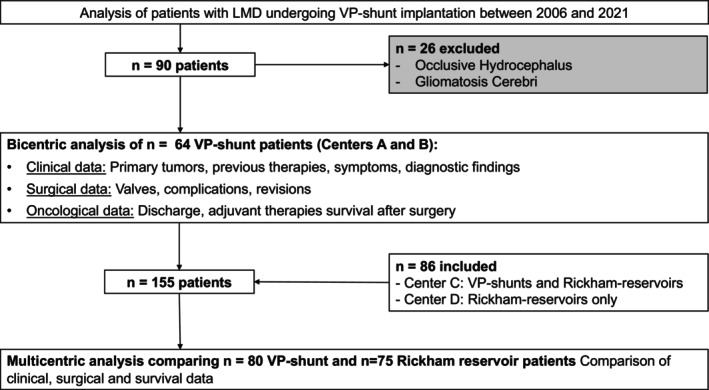
Study flow chart. The Bicentric analysis was carried out on data from Centers A and B that implant ventriculoperitoneal shunts (VPS) in patients with symptomatic intracranial hypertension and leptomeningeal disease. Center C employs both Rickham Reservoirs (RR) and VPS, and Center D implants only RR.

**FIGURE 2:**
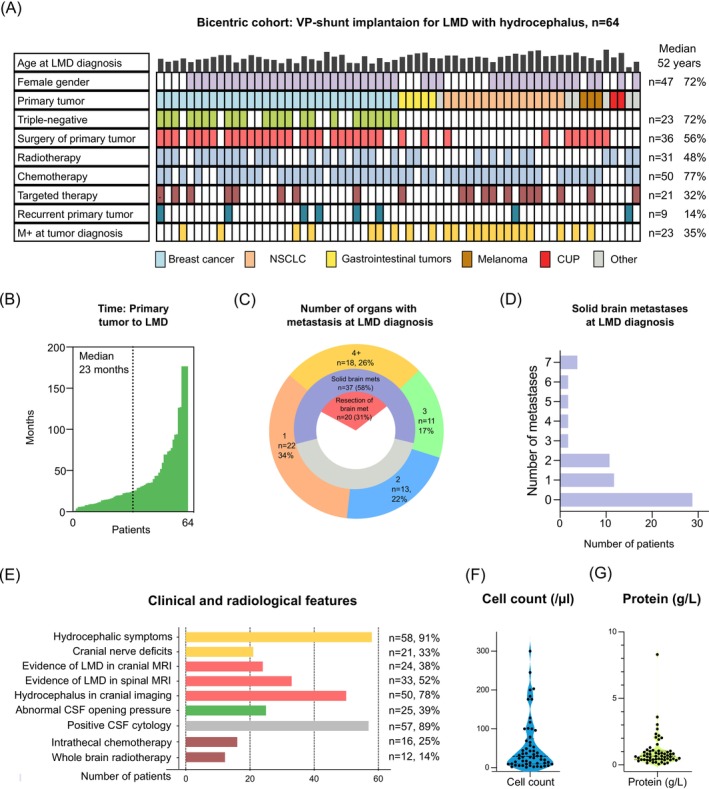
Patient characteristics of the bicentric cohort prior to ventriculoperitoneal shunt (VPS) implantation. (A) Onco‐plot of selected variables of the 64 patients with leptomeningeal disease (LMD) included in the bicentric analysis. Rows represent variables, and each column represents one patient. M + = Metastasis of primary tumor to another organ system at initial diagnosis. CUP, Cancer of Unknown Primary; GI‐Tumors, Gastrointestinal tumors; NSCLC = Non‐small Cell Lung Cancer. All therapies included pertain to the primary tumor and not LMD. (B) Elapsed time (days) from primary tumor to LMD diagnosis in *n* = 64 patients. (C) Stacked pie charts showing the number of patients with 1, 2, 3 or more organs with tumor metastases at LMD diagnosis (outer circle), the number of patients with solid brain metastases (=mets, middle circle), and the overall percentage of patients that had received a craniotomy for resection of brain metastases (inner circle). (D) Number of solid brain metastases in the bicentric cohort of 64 patients receiving VPS. (E) Clinical and radiological features of the patients in the bicentric cohort before shunting. CSF, cerebrospinal fluid; MRI, magnetic resonance imaging. (F) Violin plot of the cell count (cells/μl) in *n* = 64 patients of the bicentric VPS cohort. G: Protein concentration (g/L) in *n* = 64 patients of the bicentric VPS cohort.

Of 81 patients receiving VPS implantation for LMD in Center A, 26 patients (32%) showed glial tumors as an underlying condition or an occlusive hydrocephalus and were thus excluded due to the different nature of extracranial oncological disease. Data of nine eligible patients receiving VPS for LMD from Center B was then pooled with these 55 patients from Center A, leaving the final number of analyzed patients at 64 in the bicentric cohort (Figure 1). In these 64 patients, the median age was 52 years (IQR 44.3–60.7 years), with 72% being female (*n* = 47). Primary tumors mainly comprised breast cancer (*n* = 32, 49%), followed by non‐small cell lung cancer (NSCLC) (*n* = 16, 25%), gastrointestinal tumors (GI‐tumors, *n* = 5, 8%) and malignant melanoma (*n* = 3, 5%). Further primary tumors included ovarian cancer and cancer of unknown primary (CUP, *n* = 2, 3% each). Interestingly, 72% of patients with breast cancer (*n* = 23) harbored triple‐negative primary tumors. At initial primary tumor diagnosis, 56% of patients (*n* = 36) had received resection surgery for primary tumors, where breast cancer patients were overrepresented (*n* = 26/32 breast cancer patients, 81% vs. 14% of NSCLC patients, *n* = 2/16, *p* < 0.001, Fischer's exact test). In addition, 31 patients had received radiotherapy (48%) and 50 patients had undergone chemotherapy (77%) for their primary extracranial tumors, in addition to targeted therapy in 21 cases (32%, Figure 2A). At initial diagnosis, only 35% of the patients in the bicentric cohort (*n* = 23) demonstrated an M+ situation of their respective tumors in the TNM classification. Recurrence or progressive disease of primary tumor had been documented in nine cases (14%). The median time between primary diagnosis and LMD diagnosis was about 2 years (23.3 months, IQR 11.2–43.4 months, Figure 2B) in this cohort. In terms of tumor staging at LMD diagnosis, metastases were found in one organ in 22 (34%), in two organs in 13 (22%), in three different organs in 11 (17%), and in four or more different organs in 18 patients (26%). Of these, 37 patients (58%) showed solid brain metastases, of which 20 patients (31%) had undergone cranial resection surgery (Figure 2C). The median number of solid brain metastases in shunted LMD patients was 1 (IQR 0–2, Figure 2D). Hydrocephalic symptoms (headache, nausea, vomiting, gait disorder, diplopia) were present in 58 patients (91%) upon LMD diagnosis. In 21 cases (33%), additional cranial nerve deficits were documented, with 24 patients showing nodular LMD in cranial MRI scans (33%). On spinal MRI scans, 33 patients (52%) also showed abnormal meningeal contrast enhancements. Interestingly, 78% of the patients (*n* = 50) showed a clear evidence of CSF circulation disorder through dilatation of ventricles on initial head scans (CT/MRI, depending on the setting of hospital admission) and only 39% (*n* = 25) had abnormal CSF opening pressure after lumbar puncture (LP). However, upon drainage of at least 30 mL of CSF, 43 patients reported symptom improvement (67%). CSF histopathology revealed positive CSF cytology in 89% of the CSF samples (*n* = 57), while in the remaining cases high clinical and radiographic suspicion of LMD led to VPS implantation. Strikingly, a CSF cell count above 5 cells/μl was found in only 52% of patients (*n* = 33, median 29 cells/μl, IQR 9–74 cells/μl) and abnormal protein in 64% (*n* = 41, median 0.65 g/L, IQR 0.4–1.043 g/L). Upon LMD diagnosis, 16 patients had received intrathecal chemotherapy (25%, eight through implanted Rickham reservoirs, with later conversion to VPS and the other half through lumbar infusion) including methotrexate in 12 cases and trastuzumab in four cases. In 12 patients (14%), whole‐brain radiotherapy was employed as an initial therapy upon detection of LMD (Figure 2E–G).

### 3.2 VPS provides symptom relief and therapy amenability in LMD patients

**FIGURE 3:**
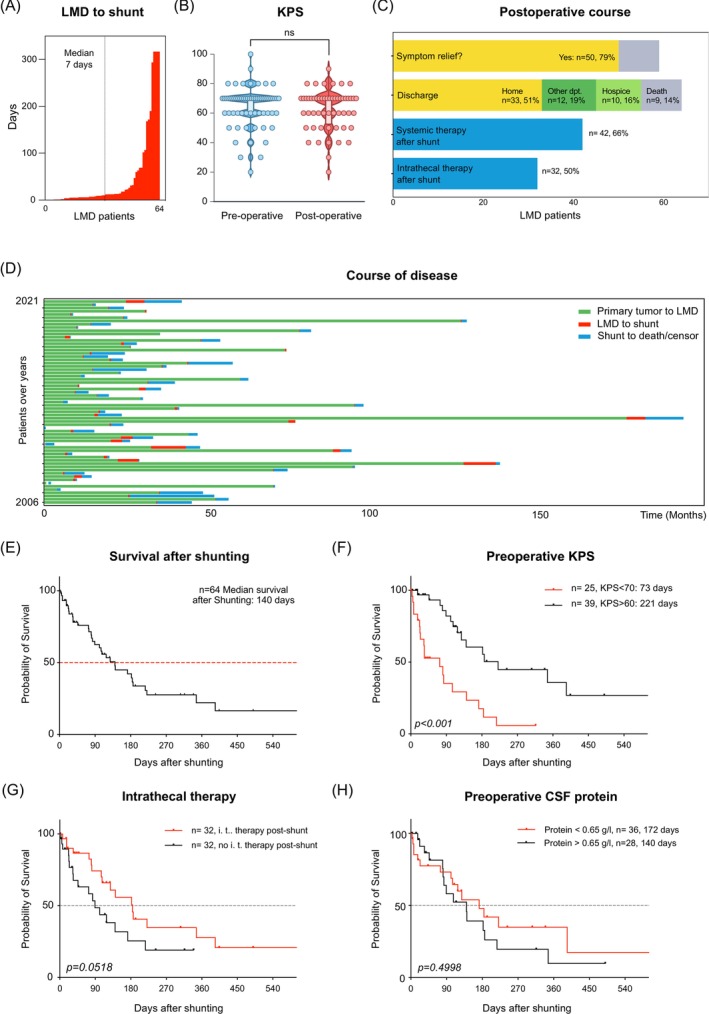
Surgical and survival outcomes of patients receiving VPS for LMD (Leptomeningeal Disease). (A) Time (days) lapsing between LMD diagnosis and VP‐shunting in the *n* = 64 patients of the bicentric cohort. (B) Violin plots of pre‐ and post‐operative Karnofsky Performance Score = KPS of VPS patients. Note that patients with in‐hospital death (*n* = 9) were excluded (ns = non‐significant, Wilcoxon matched‐pairs signed rank test). (C) Overview of the postoperative course of VPS patients, including rate of symptom relief, post‐operative discharge (also to other departments = dept.) and further systemic and intrathecal therapy. (D) Swimmer's plot of the course of disease (across) in *n* = 64 patients (down, chronologically ordered from 2006 to 2021) in the bicentric cohort highlighting the time (in months) from primary tumor diagnosis to LMD (green), from LMD to shunt (red) and from shunting to death (or censorship, blue). (E) Survival of LMD patients after VP‐shunting. (F) Survival (days) in patients after VPS (*n* = 64) with stratification based on Karnofsky Performance Score (KPS < 70 vs. KPS > 60 (*p* < 0.001, Log‐rank [Mantel‐Cox] test). (G) Survival after shunting in patients receiving postoperative intrathecal therapy (i.t.) compared to patients without postoperative intrathecal therapy (*p* = 0.0518, Log‐rank [Mantel‐Cox] test). (H) Survival of patients with preoperative CSF protein concentrations higher than the median in this cohort (0.65 g/L) compared to patients with lower protein concentrations (*p* = 0.4998, Log‐rank [Mantel‐Cox] test).

In the bicentric cohort, the median time between LMD diagnosis to shunting was 7 days (IQR 5–23 days), with the maximum being 317 days (Figure 3A). After VPS insertion, there was no significant difference in the KPS of patients surviving surgery at discharge compared to their pre‐operative KPS (pre‐operative 65% vs. post‐operative 62%, *p* = 0.0853, Wilcoxon matched‐pairs signed rank test). To provide a meaningful conclusion over the ‘real‐life’ KPS, deceased patients were excluded (Figure 3B). 79% of patients (*n* = 50) reported improvement of their symptoms, with 51% discharged home (*n* = 33), and 19% (*n* = 12) discharged to another department for further therapy. Indeed, 42 patients (66%) underwent systemic therapy after VPS implantation, with 32 patients (50%) undergoing intrathecal therapy. In 10 cases (16%), patients were transferred to a hospice after the procedure, while nine patients (14%) died during their hospital stay (Figure 3C), which was related to surgery in only one case (see Table 1), Figure 3D provides an overview of the course of disease of the patients included in the bicentric cohort. The valves implanted varied over time and included programmable valves in 50 cases (79%, Table 2). An on–off valve was additionally implanted in 29 cases (45%). In 12 patients (19%), a Codman® Certas® Plus Programmable Valve was implanted and set to pressure level ‘8’ = ‘virtual off’ during intrathecal therapy. The surgical complication rate was 25% (*n* = 16), including valve dysfunction (*n* = 6), catheter malposition (abdominal *n* = 2, ventricular *n* = 1), shunt infection or superficial wound healing disorders (*n* = 2 each), and hygroma or postoperative hemorrhages requiring neurosurgical intervention (Table 2). In this bicentric VPS cohort, the median overall survival (OS) of LMD patients after shunting was 4.7 months (140 days, Figure 3E). Out of 64 patients, 30 were censored in the survival analysis due to loss‐to‐follow‐up. Patients with a Karnofsky Performance Score (KPS) >60 showed a significantly longer OS after shunting than patients with KPS <70 (*p* < 0.001, Log‐rank [Mantel‐Cox] test, Figure 3F). There was a statistically non‐significant survival benefit in patients receiving postoperative intrathecal therapy (median OS of 184 days in the intrathecal therapy subgroup vs. 89 days in patients without, *p* = 0.0518, Log‐rank [Mantel‐Cox] test, Figure 3G) or depending on hormonal status (Estrogen receptor positive/Progesterone receptor positive and/or Human Epidermal Growth Factor receptor 2 positive) OS = 170 days vs. triple‐negative breast cancer OS = 128 days, *p* = 0.180 (Log‐rank [Mantel‐Cox] test), Figure S1. No difference in survival was noted in patients with higher CSF protein concentrations (172 days; the median CSF protein concentration of 0.65 g/L was used a threshold), compared to patients with lower protein concentrations (140 days, *p* = 0.4998, Log‐rank [Mantel‐Cox] test, Figure 3H).

**TABLE 1:** Medical complications leading to death of Center A and B patients receiving ventriculoperitoneal shunts (VPS) for leptomeningeal disease with malresorptive hydrocephalus.

Complication leading to death	*n* (%)
Extracranial infection	3 (5%)
Chemotherapy related agranulocytosis	2 (3%)
Neurological deterioration through progress of LMD	3 (5%)
Shunt‐related	1 (2%)
Total	9 (19%)

**TABLE 2:** Implanted valves with corresponding complications leading to surgical revision of Center A and B patients receiving ventriculoperitoneal shunts (VPS) for leptomeningeal disease with malresorptive hydrocephalus.

Valve	*n*	%	Surgical complications (*n* = 16, 25%)										
			Valve dysfunction		Cather complication^a^		Infection		Wound healing disorder		Hygroma/SDH^b^		Total
Frontal VPS *n* = 64 + On–Off *n* = 29 (45%)			*n*	%^c^	*n*	%^c^	*n*	%^c^	*n*	%^c^	*n*	%^c^	
Integra	8	13%	1	6%	0	0%	0	0%	0	0%	1	6%	2
Hakim	8	13%	2	13%	0	0%	0	0%	1	6%	0	0%	3
Certas Plus	12	19%	2	13%	2	13%	1	6%	0	0%	1	6%	6
Dual‐Switch	6	9%	0	0%	0	0%	1	6%	1	6%	0	0%	2
PROGAV 1.0	21	33%	1	6%	1	6%	0	0%	0	0%	1	6%	3
PROGAV 2.0	7	11%	0	0%	0	0%	0	0%	0	0%	0	0%	0
PaediGAV	2	3%	0	0%	0	0%	0	0%	0	0%	0	0%	0
Total	64	100%	6	38%	3	19%	2	13%	2	13%	3	19%	16
*p*‐value for valve^d^			0.5393		0.6482		0.3318		0.1761		0.9395		0.159

*Note*: Hakim = Medos‐Hakim valve.

^a^ Occlusion of ventricular catheter (*n* = 1) and dislocation of abdominal catheter (*n* = 2).

^b^ Leading to death in one case.

^c^ Rate of complication from total complications (*n* = 16).

^d^ Fisher's exact test for overrepresentation of a particular valve in any complication group.

### 3.3 Multicentric analysis comparing VPS and RR in LMC patients with hydrocephalus

**FIGURE 4:**
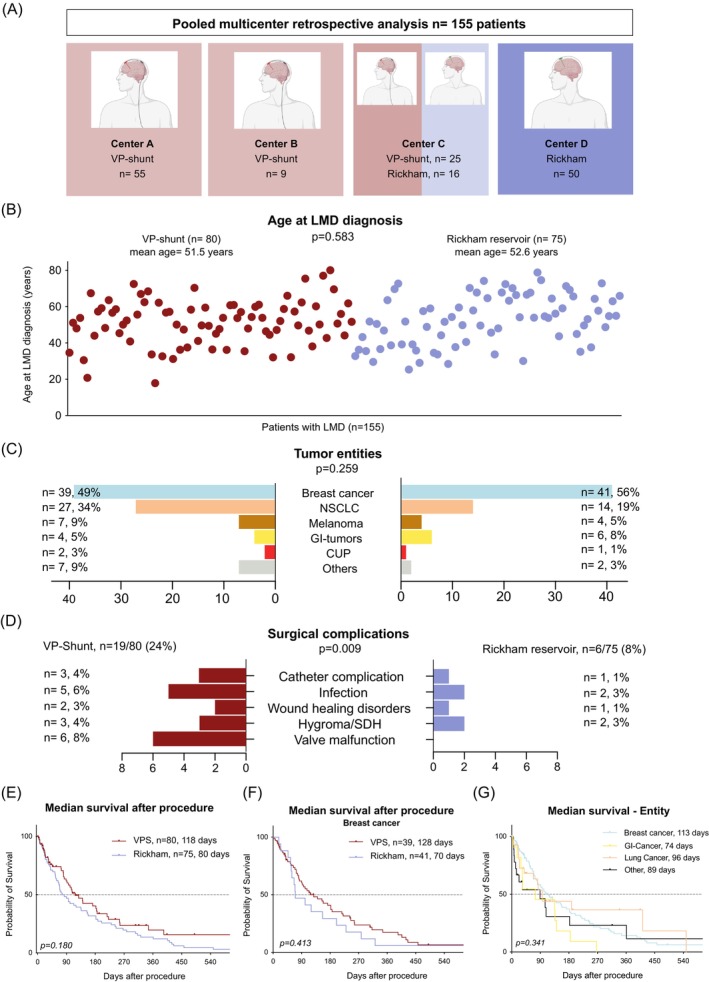
Pooled multicenter retrospective analysis of Ventriculoperitoneal shunt (VPS) and Rickham reservoir (RR) patients. (A) Overview of the number of patients with the respective procedure performed included from each center. (B) Plot of patient age at LMD diagnosis showing no differences between the VPS (left, red) and RR cohorts (right, violet, two‐tailed Student's *t* test). (C) Bar chart comparing proportions of primary tumor entities in VPS and RR patients (chi‐square test). CUP, Cancer of Unknown Primary; GI‐Tumors, Gastrointestinal tumors; NSCLC, Non‐small Cell Lung Cancer. (D) Comparison of complications requiring surgical revision after VPS vs. after RR placement (Fisher's exact test). SDH = Subdural Hematoma. (E) Survival of LMD patients after VP‐shunting (*n* = 80) vs. RR placement (*n* = 75), *p* = 0.180 (Log‐rank (Mantel‐Cox) test). (F) Survival (days) in patients with breast cancer as a primary tumor after VPS (*n* = 39) vs. RR (*n* = 41), *p* = 0.431 (Log‐rank (Mantel‐Cox) test). (G) Survival (days) stratified according to primary neoplasm, *p* = 0.341 (Log‐rank [Mantel‐Cox] test).

After establishing that VPS implantation could provide an acceptable symptom relief under a reasonable perioperative risk and, given the high rate of patients receiving an intrathecal therapy over the implanted reservoir of the shunt in our cohort, we aimed to determine whether a Rickham reservoir (RR) implantation could be a viable alternative to VPS. To this end, we collected data from two further centers: In one, Center C (VPS: *n* = 25, RR: *n* = 16), both procedures are applied, depending on the clinical presentation of the patient. In Center D (RR: *n* = 50), no VPS implantations are carried out, but rather RR implantation with CSF drainage as required and intrathecal therapy administration via reservoir tapping. Altogether, we compared *n* = 80 patients with VPS and *n* = 75 patients with RR in this multicentric retrospective analysis (Figure 4A). In both groups, the median age of patients at LMD diagnosis was similar (51.5 years for VPS vs. 52.6 years for RR patients, *p* = 0.583, two‐tailed Student's *t* test, Figure 4B) and the primary tumor entities were comparable (mostly breast cancer: *n* = 39, 49% vs. *n* = 41, 56% and lung cancer: *n* = 27, 34% vs. *n* = 14, 19% in VPS vs. RR patients, *p* = 0.259, Chi‐square test, Figure 4C). We found the rate of complications leading to surgical revision to be significantly higher in the VPS than the RR group (*n* = 19, 24% vs. *n* = 6, 8%, *p* = 0.0088, Fisher's exact test). This was mainly driven by valve related complications (n = 6 valve malfunction/occlusion, accounting for 31% of the total complication rate in the VPS group, Figure 4D). Failure of further shunt components included malposition of the abdominal catheter (*n* = 2 in the VPS group). The median initial protein level in patients with valve dysfunction was 0.4 g/L, which is lower than the median of the core shunt cohort with 0.65 g/L. There was no significant difference in the rate of infection between both groups (*n* = 5, 6% vs. *n* = 2, 3%, *p* = 0.444, Fisher's exact test, Figure 4D). The rate of RR to VPS conversion in this cohort was 3% (*n* = 2/75). We compared the overall survival (OS) rates of patients in both groups to elucidate whether this higher complication rate is mitigated by or the consequence of a longer survival of LMD patients. Indeed, there was no significant difference in the median OS between both groups, although VPS patients showed a longer OS (118 days vs. 80 days in the RR group, Log‐rank (Mantel‐Cox) test, Figure 4E). We then compared a relatively ‘clean’ sub‐cohort containing breast cancer patients only, to rule out possible confounding effects due to eventual disproportions of primary tumor disease. Although differences were noticed in the OS of both patient subgroups (VPS breast cancer patients 128 days vs. RR breast cancer patients 70 days), they were not significant (*p* = 0.431, Log‐rank [Mantel‐Cox] test, Figure 4F). When stratified by primary diagnosis, breast cancer patients had a longer but non‐significant median survival after the procedure (128 days) compared to those with lung cancer (96 days), gastrointestinal tumors (74 days), or the heterogeneous remainder of the cohort (89 days, *p* = 0.341, Log‐rank [Mantel‐Cox] test, Figure 4G). Altogether, the data show a higher complication rate of VPS but cannot establish longer survival after the procedure compared to RR in LMD patients.

## 4 DISCUSSION

LMD is regarded as a condition of highly palliative nature with poor prognosis.^3^ However, in the wake of improved survival in the most affected tumor entities (breast and lung cancer), hydrocephalus secondary to LMD is of utmost clinical relevance, making CSF diversion indispensable in a specific subgroup of affected patients.^31^, ^32^ Currently, there is little robust data that informs on the optimal modality of hydrocephalus management in affected patients, especially when intrathecal therapy is planned,^16^ apart from the ESMO‐EANO guideline recommendation of palliative VPS implantation in patients with hydrocephalus and LMD.^14^

In this multicenter analysis, we found no difference in the median OS of affected patients after VPS or RR implantation for ventricular access, under comparable patient age and distribution of primary tumors, even after stratifying for patients with the same primary disease (breast cancer). Indeed, at the same time, RR placement was associated with a significantly lower complication rate compared to VPS implantation, primarily driven by valve malfunction. This is the first study directly comparing both modalities and integrating data from different European neurosurgical centers in the context of LMD.

A vital aspect in the management of hydrocephalus in LMD is neurological symptom relief, which was achieved in about 80% of the patients with VPS, concordant to previous reports.^33^ An analysis of pre‐ and post‐operative KPS in our cohort showed that VPS patients maintain a steady KPS after surgery (if not deceased), and hence retain amenability for intrathecal therapy. In addition, about 70% of the patients involved were (made) amenable to further therapy, undergoing post‐shunting systemic therapy in 66% and intrathecal therapy in 50% of cases. However, the higher VPS complication rate justifies considering the alternative of RR implantation with multiple CSF drainages under intrathecal therapy, even for patients with hydrocephalus, especially given similar survival and lower complication rates of the RR patient subgroups in the multicentric retrospective analysis. Our findings do not support the premise that patients with RR and planned intrathecal therapy necessarily require CSF drainage during treatment or that they can avoid additional hospital visits for drainage. This assumption remains hypothetical. RR placement might lead to more frequent hospital visits, potentially offsetting its presumed advantage of fewer complications compared to VPS. However, if CSF drainage is required beyond intrathecal therapy, a second short procedure for permanent peritoneal diversion could be a reasonable option. The likelihood of requiring an additional surgery would not necessarily be higher for RR patients, as the risk is comparable to the real‐life revision rate for VPS (24%). Reported data on both complication rates and types support this perspective.^4^, ^18^, ^34^ The valve failure rate in VPS patients, the most common complication in this cohort, was 9%. However, we found no association between preoperative CSF protein levels and valve malfunctions. In fact, protein levels in patients with valve failure were, on average, lower than in the rest of the cohort. This observation aligns with previous reports on LMD patients, further suggesting that high CSF protein levels might not be linked to shunt complications.^4^

The median post‐surgery survival of patients after VPS placement in the bicentric analysis of this study was about 5 months, which is comparable to what has been described in the past.^4^, ^20^, ^35^ Other studies had reported median survival rates of longer than 7 months in selected patients.^17^, ^36^ We noticed a high rate of patients with triple‐negative breast cancer, which is reported to be much lower in previous patient cohorts,^37^ underscoring the heterogeneity of patients with LMD and the importance of patient stratification in this context.

Because this cohort is dominated by breast and lung cancer patients, caution should be practiced when generalizing its findings to LMD patients with other primary diseases. Importantly, intrathecal therapy after VP‐shunting showed a trend towards longer survival, with the difference being non‐significant in this cohort; the potential effect of intrathecal therapy after VPS or RR surgery on OS could pose a bias to the comparability of both modalities altogether. In more than half of the VPS cohort, surgical resection for cranial metastases had been performed prior to LMD diagnosis. It is unclear whether tumor dissemination to the CSF could be influenced by surgical factors such as a juxta‐ventricular localization, opening of the lateral ventricle, or surgery itself. A recent study on glial tumors found no association between the act of opening the lateral ventricles and an elevated risk of dissemination or hydrocephalus in patients with high‐grade glioma,^38^ although conflicting evidence is found in the literature.^39^, ^40^

Interestingly, the rate of symptom improvement after lumbar tapping of patients in the bicentric cohort was only 67%, questioning whether lumbar tapping or CSF opening pressure (which was pathological in only about 40% of patients) can solely predict the worthwhileness of the VPS implantation, especially given the fact that ventricular intrathecal therapies in patients with confirmed or probable LMD would justify CSF access anyway.^14^, ^41^

Although VPS implantation has been often considered ‘ethically controversial’, our study contributes to the growing body of evidence indicating that VPS can effectively relieve symptoms in patients with LMD. Moreover, these patients can achieve sufficient post‐surgery survival to receive intrathecal and systemic therapy, with a complication rate comparable to that of other VPS cohorts of heterogenous surgical indications.^42^, ^43^ On the other hand, the high in‐hospital mortality rate (14%) underscores the palliative nature of this disease, although only one of the deaths documented was a direct consequence of surgery; the remaining cases were rather associated with the course of the primary disease or with complications related to chemotherapy. However, it should be emphasized that despite a 24% revision surgery rate, many LMD patients with hydrocephalus remain suitable candidates for primary VPS implantation—for instance, those requiring frequent pre‐shunting lumbar punctures or those with strictly palliative indications. Furthermore, in light of our data, offering a sub‐cohort of LMD patients with hydrocephalus the choice between both modalities during consultations could be justifiable.

## 5 LIMITATIONS

This study has several limitations, let alone its retrospective nature. Because only patients receiving CSF drainage for intraventricular therapy were included, this study introduces a clear selection bias and cannot hence reflect the clinical course of all patients with LMD. This is also evident through the eventuality of a stark inter‐center disparity both pertaining to the selection bias and to the diverse post‐operative treatment modalities that could have varied between centers and cannot be controlled for in this setting. In this regard, interpreting the findings of this study in the context of LMD should be limited to patients with hydrocephalus. Similarly, there is an inherent selection bias in patients undergoing VPS, as only those with a KPS >60—also reflected in this cohort—are typically considered for surgery. Consequently, post‐operative KPS cannot fully capture potential improvements in quality of life. At best, it may indicate that surgery does not necessarily enhance overall functional status, even though it alleviates some symptoms in a disease that is otherwise progressively debilitating. Because of the low number of study subjects in the bicentric cohort, we collected patient data from further institutions and added an analysis comparing VP‐shunt patients to Rickham reservoir patients. However, the sample size remains smaller than what would be required to infer on patient survival based on stratification, especially with the heterogeneity of patients affected with the disease at hand. We still did not observe significant differences in survival between the two modalities in a ‘clean’ cohort of breast cancer patients. However, the relatively large survival difference between the groups suggests a potential risk of overfitting in our statistical model. Owing to the nature of this patient cohort and the high number of hospice discharges, many patients with VPS were censored due to loss to follow‐up in the analysis, making the interpretation of survival data challenging. This is also true for RR patients, where long‐term follow‐up data is mostly not available due to the retrospective nature of this analysis, undermining a ‘real’ comparison of the post‐operative quality of life between both cohorts.

## 6 CONCLUSION

While surgical CSF drainage in patients with LMD relieves symptoms of intracranial hypertension in most cases, median survival remains under 5 months. Compared to RR implantation, VPS bears higher rates of complications and revisions without a clear survival benefit. Nevertheless, despite low OS rates and differences in complication rates, differential therapeutic evaluations including shunting are subject to individual and palliative nature.

## AUTHOR CONTRIBUTIONS

**Obada T. Alhalabi:** Conceptualization; investigation; methodology; validation; visualization; writing – review and editing; project administration; data curation; writing – original draft. **Lukas Klein:** Visualization; formal analysis; data curation. **David Wasilewski:** Data curation. **Amine Mellal:** Writing – review and editing; investigation. **Carmen Büsken:** Data curation; visualization. **Clara Buszello:** Data curation; formal analysis. **Giulia Cossu:** Investigation; validation. **Ilker Y. Eyüpoglu:** Conceptualization; resources; supervision. **Andreas W. Unterberg:** Supervision; resources. **Peter Vajkoczy:** Supervision; resources. **Gabriele Schackert:** Supervision; resources. **Mahmoud Messerer:** Project administration; data curation; supervision. **Martin Misch:** Data curation; supervision; formal analysis. **Tobias Kessler:** Data curation; supervision; formal analysis. **Wolfgang Wick:** Supervision; resources; project administration; writing – review and editing. **Christine Jungk:** Data curation; supervision; writing – review and editing. **Ahmed El Damaty:** Conceptualization; project administration; resources. **Sandro M. Krieg:** Conceptualization; data curation; supervision. **Tareq A. Juratli:** Conceptualization; supervision; resources. **Alexander Younsi:** Conceptualization; investigation; writing – review and editing; project administration; formal analysis; supervision; resources.

## CONFLICT OF INTEREST STATEMENT

The authors declare that the research was conducted in the absence of any commercial or financial relationships that could be construed as a potential conflict of interest. Tareq A. Juratli reports honoraria from CSL‐Behring, not related to the topic.

## ETHICS STATEMENT

The local standing committee of ethical practice approved the protocol of this retrospective analysis (file number Heidelberg: S‐084/2022, Berlin: EA1/292/23, Lausanne: CER‐VD 2024‐00866, Dresden: EK323122008) which was performed in accordance with the ethical standards laid down in the 1964 Declaration of Helsinki and its later amendments, and for which patient consent was waived.

## Supporting information


**DATA S1.** Supporting Information.

## Data Availability

The data that support the findings of this study are available from the corresponding author upon reasonable request.

## References

[1] Gleissner B , Chamberlain MC . Neoplastic meningitis. Lancet Neurol. 2006;5:443‐452. 16632315 10.1016/S1474-4422(06)70443-4

[2] Le Rhun E , Taillibert S , Chamberlain MC . Carcinomatous meningitis: leptomeningeal metastases in solid tumors. Surg Neurol Int. 2013;4:S265‐S288. 23717798 10.4103/2152-7806.111304 PMC3656567

[3] Glantz MJ , Jaeckle KA , Chamberlain MC , et al. A randomized controlled trial comparing intrathecal sustained‐release cytarabine (DepoCyt) to intrathecal methotrexate in patients with neoplastic meningitis from solid tumors. Clin Cancer Res. 1999;5:3394‐3402. 10589750

[4] Bander ED , Yuan M , Reiner AS , et al. Cerebrospinal fluid diversion for leptomeningeal metastasis: palliative, procedural and oncologic outcomes. J Neurooncol. 2021;154:301‐313. 34406564 10.1007/s11060-021-03827-2 PMC8504535

[5] Wallace G , Kundalia R , Vallebuona E , et al. Factors associated with overall survival in breast cancer patients with leptomeningeal disease (LMD): a single institutional retrospective review. Breast Cancer Res. 2024;26:55. 38553702 10.1186/s13058-024-01789-7 PMC10979566

[6] Nguyen A , Nguyen A , Dada OT , et al. Leptomeningeal metastasis: a review of the pathophysiology, diagnostic methodology, and therapeutic landscape. Curr Oncol. 2023;30:5906‐5931. 37366925 10.3390/curroncol30060442 PMC10297027

[7] de Oca M , Delgado M , Cacho Díaz B , et al. The comparative treatment of intraventricular chemotherapy by Ommaya reservoir vs. lumbar puncture in patients with leptomeningeal carcinomatosis. Front Oncol. 2018;8:509. 30524956 10.3389/fonc.2018.00509 PMC6256195

[8] Gwak HS , Joo J , Kim S , et al. Analysis of treatment outcomes of intraventricular chemotherapy in 105 patients for leptomeningeal carcinomatosis from non‐small‐cell lung cancer. J Thorac Oncol. 2013;8:599‐605. 23422833 10.1097/JTO.0b013e318287c943

[9] Glitza Oliva IC , Ferguson SD , Bassett R , et al. Concurrent intrathecal and intravenous nivolumab in leptomeningeal disease: phase 1 trial interim results. Nat Med. 2023;29:898‐905. 36997799 10.1038/s41591-022-02170-x PMC10115650

[10] Roy‐O'Reilly MA , Lanman T , Ruiz A , Rogawski D , Stocksdale B , Nagpal S . Diagnostic and therapeutic updates in leptomeningeal disease. Curr Oncol Rep. 2023;25:937‐950. 37256537 10.1007/s11912-023-01432-2 PMC10326117

[11] Buszek SM , Chung C . Radiotherapy in leptomeningeal disease: a systematic review of randomized and non‐randomized trials. Front Oncol. 2019;9:1224. 31803614 10.3389/fonc.2019.01224 PMC6872542

[12] Ferguson SD , Bindal S , Bassett RL Jr , et al. Predictors of survival in metastatic melanoma patients with leptomeningeal disease (LMD). J Neurooncol. 2019;142:499‐509. 30847840 10.1007/s11060-019-03121-2

[13] Arvold ND , Lee EQ , Mehta MP , et al. Updates in the management of brain metastases. Neuro Oncol. 2016;18:1043‐1065. 27382120 10.1093/neuonc/now127 PMC4933491

[14] Le Rhun E , Weller M , van den Bent M , et al. Leptomeningeal metastasis from solid tumours: EANO‐ESMO clinical practice guideline for diagnosis, treatment and follow‐up. ESMO Open. 2023;8:101624. 37863528 10.1016/j.esmoop.2023.101624 PMC10619142

[15] Grossman SA , Krabak MJ . Leptomeningeal carcinomatosis. Cancer Treat Rev. 1999;25:103‐119. 10395835 10.1053/ctrv.1999.0119

[16] Lamba N , Fick T , Nandoe Tewarie R , Broekman ML . Management of hydrocephalus in patients with leptomeningeal metastases: an ethical approach to decision‐making. J Neurooncol. 2018;140:5‐13. 30022283 10.1007/s11060-018-2949-7 PMC6182391

[17] Mitsuya K , Nakasu Y , Hayashi N , et al. Palliative cerebrospinal fluid shunting for leptomeningeal metastasis‐related hydrocephalus in patients with lung adenocarcinoma: a single‐center retrospective study. PLoS One. 2019;14:e0210074. 30629621 10.1371/journal.pone.0210074 PMC6328154

[18] Su YH , Chiang CL , Yang HC , et al. Cerebrospinal fluid diversion and outcomes for lung cancer patients with leptomeningeal carcinomatosis. Acta Neurochir. 2022;164:459‐467. 33646444 10.1007/s00701-021-04763-w

[19] Jung TY , Chung WK , Oh IJ . The prognostic significance of surgically treated hydrocephalus in leptomeningeal metastases. Clin Neurol Neurosurg. 2014;119:80‐83. 24635931 10.1016/j.clineuro.2014.01.023

[20] Kim HS , Park JB , Gwak HS , Kwon JW , Shin SH , Yoo H . Clinical outcome of cerebrospinal fluid shunts in patients with leptomeningeal carcinomatosis. World J Surg Oncol. 2019;17:59. 30917830 10.1186/s12957-019-1595-7 PMC6438037

[21] Boogerd W , van den Bent MJ , Koehler PJ , et al. The relevance of intraventricular chemotherapy for leptomeningeal metastasis in breast cancer: a randomised study. Eur J Cancer. 2004;40:2726‐2733. 15571954 10.1016/j.ejca.2004.08.012

[22] Schneider M , Wispel C , Potthoff A‐L , et al. Patients with leptomeningeal carcinomatosis and hydrocephalus‐feasibility of combined ventriculoperitoneal shunt and reservoir insertion for intrathecal chemotherapy. Curr Oncol. 2024;31:2410‐2419. 38785461 10.3390/curroncol31050180 PMC11120415

[23] Murakami Y , Ichikawa M , Bakhit M , et al. Palliative shunt surgery for patients with leptomeningeal metastasis. Clin Neurol Neurosurg. 2018;168:175‐178. 29567579 10.1016/j.clineuro.2018.03.008

[24] Abouharb S , Ensor J , Loghin ME , et al. Leptomeningeal disease and breast cancer: the importance of tumor subtype. Breast Cancer Res Treat. 2014;146:477‐486. 25038877 10.1007/s10549-014-3054-z

[25] Morikawa A , de Stanchina E , Pentsova E , et al. Phase I study of intermittent high‐dose lapatinib alternating with capecitabine for HER2‐positive breast cancer patients with central nervous system metastases. Clin Cancer Res. 2019;25:3784‐3792. 30988080 10.1158/1078-0432.CCR-18-3502 PMC6773251

[26] Ahn MJ , Chiu CH , Cheng Y , et al. Osimertinib for patients with leptomeningeal metastases associated with EGFR T790M‐positive advanced NSCLC: the AURA leptomeningeal metastases analysis. J Thorac Oncol. 2020;15:637‐648. 31887431 10.1016/j.jtho.2019.12.113

[27] Lee J , Choi Y , Han J , et al. Osimertinib improves overall survival in patients with EGFR‐mutated NSCLC with leptomeningeal metastases regardless of T790M mutational status. J Thorac Oncol. 2020;15:1758‐1766. 32652216 10.1016/j.jtho.2020.06.018

[28] Mashiach E , Alzate JD , De Nigris Vasconcellos F , et al. Long‐term survival from breast cancer brain metastases in the era of modern systemic therapies. Neurosurgery. 2024;94:154‐164. 37581437 10.1227/neu.0000000000002640

[29] Sandberg DI , Bilsky MH , Souweidane MM , Bzdil J , Gutin PH . Ommaya reservoirs for the treatment of leptomeningeal metastases. Neurosurgery. 2000;47:49‐54. 10917346 10.1097/00006123-200007000-00011

[30] Paff M , Alexandru‐Abrams D , Muhonen M , Loudon W . Ventriculoperitoneal shunt complications: a review. Interdiscip Neurosurg. 2018;13:66‐70.

[31] Guo F , Kuo YF , Shih YCT , Giordano SH , Berenson AB . Trends in breast cancer mortality by stage at diagnosis among young women in the United States. Cancer. 2018;124:3500‐3509. 30189117 10.1002/cncr.31638 PMC6191354

[32] Thandra KC , Barsouk A , Saginala K , Aluru JS , Barsouk A . Epidemiology of Lung Cancer. Contemp Oncol (Pozn). 2021;25:45‐52. 33911981 10.5114/wo.2021.103829 PMC8063897

[33] Lin N , Dunn IF , Glantz M , et al. Benefit of ventriculoperitoneal cerebrospinal fluid shunting and intrathecal chemotherapy in neoplastic meningitis: a retrospective, case‐controlled study. J Neurosurg. 2011;115:730‐736. 21721878 10.3171/2011.5.JNS101768

[34] Dhaliwal J , Ruiz‐Perez M , Mihaela‐Vasilica A , Chari A , Hill CS , Thorne L . Survival and quality of life after CSF diversion in adult patients with leptomeningeal metastasis–associated hydrocephalus: a systematic review and meta‐analysis. Neurosurg Focus. 2023;55:E16. 10.3171/2023.5.FOCUS23195 37527677

[35] Lu VM , Abou‐Al‐Shaar H , Bin‐Alamer O , Luther EM , Benjamin CG . Postoperative course of cerebrospinal fluid diversion in the setting of leptomeningeal disease: a systematic review, meta‐analysis, and meta‐regression with an illustrative case. J Neurooncol. 2023;163:29‐37. 37191912 10.1007/s11060-023-04334-2

[36] Franzoi MA , Hortobagyi GN . Leptomeningeal carcinomatosis in patients with breast cancer. Crit Rev Oncol Hematol. 2019;135:85‐94. 30819451 10.1016/j.critrevonc.2019.01.020

[37] Griguolo G , Pouderoux S , Dieci MV , et al. Clinicopathological and treatment‐associated prognostic factors in patients with breast cancer leptomeningeal metastases in relation to tumor biology. Oncologist. 2018;23:1289‐1299. 30120164 10.1634/theoncologist.2018-0200 PMC6291333

[38] Cofano F , Bianconi A , De Marco R , et al. The impact of lateral ventricular opening in the resection of newly diagnosed high‐grade gliomas: a single center experience. Cancers (Basel). 2024;16:1574. 38672655 10.3390/cancers16081574 PMC11049264

[39] Lowe SR , Wang CP , Brisco A , et al. Surgical and anatomic factors predict development of leptomeningeal disease in patients with melanoma brain metastases. Neuro Oncol. 2022;24:1307‐1317. 35092434 10.1093/neuonc/noac023 PMC9340645

[40] Morshed RA , Saggi S , Cummins DD , et al. Identification of risk factors associated with leptomeningeal disease after resection of brain metastases. J Neurosurg. 2023;139:402‐413. 36640095 10.3171/2022.12.JNS221490 PMC11208084

[41] le Rhun E , Devos P , Weller J , et al. Prognostic validation and clinical implications of the EANO ESMO classification of leptomeningeal metastasis from solid tumors. Neuro Oncol. 2021;23:1100‐1112. 33367859 10.1093/neuonc/noaa298 PMC8301235

[42] Volkov AA , Filis AK , Vrionis FD . Surgical treatment for leptomeningeal disease. Cancer Control. 2017;24:47‐53. 28178712 10.1177/107327481702400107

[43] Lin N , Dunn IF , Glantz M , et al. Benefit of ventriculoperitoneal cerebrospinal fluid shunting and intrathecal chemotherapy in neoplastic meningitis: a retrospective, case‐controlled study: clinical article. J Neurosurg. 2011;115:730‐736. 21721878 10.3171/2011.5.JNS101768

