# Supplementary material for: Managing hydrocephalus in patients with leptomeningeal disease: A multicenter retrospective analysis
Source: Int J Cancer. 2025 Jun 4;157(8):1613–24. doi: 10.1002/ijc.35505 (PMC12375857; doi:10.1002/ijc.35505)
Supplement: Supplementary file 1 — DATA S1. Supporting Information. [file IJC-157-1613-s001.pdf]

**Managing Hydrocephalus in patients with leptomeningeal disease: A multicenter retrospective analysis**

Obada T. Alhalabi, Lukas Klein, David Wasilewski, Amine Mellal, Carmen Büsken, Clara Buszello, Giulia Cossu, Ilker Y. Eyüpoglu, Andreas W. Unterberg, Peter Vajkoczy, Gabriele Schackert, Mahmoud Messerer, Martin Misch, Tobias Kessler, Wolfgang Wick, Christine Jungk, Ahmed El Damaty, Sandro M. Krieg, Tareq A. Juratli, Alexander Younsi.

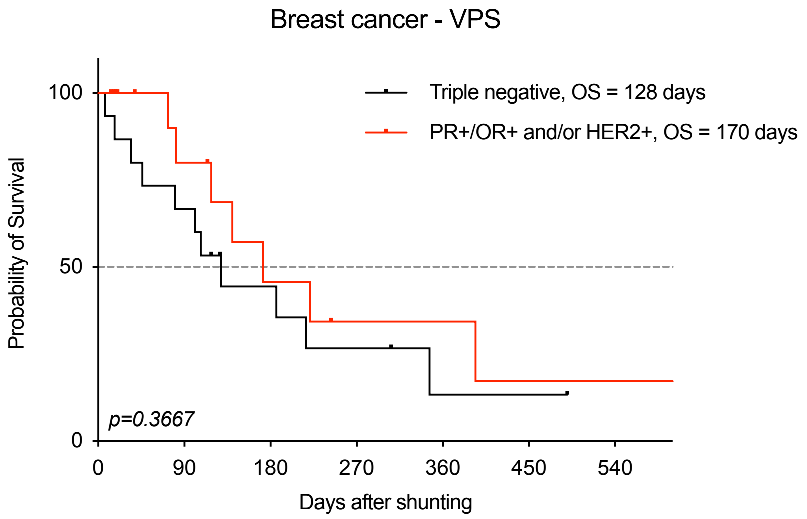


**Supplementary Figure 1:** Kaplan Meier curves with median overall Survival (OS) of LMD patients with breast cancer after VP-shunting (VPS, n=32) depending on hormone status (ER+=Estrogen receptor positive/Progesterone receptor positive= PR+, Human Epidermal Growth Factor Receptor 2 positive= HER2+, p=0.180 (Log-rank (Mantel-Cox) test).
